# Linguistic features of fragrances: The role of grammatical gender and gender associations

**DOI:** 10.3758/s13414-019-01729-0

**Published:** 2019-05-01

**Authors:** Laura J. Speed, Asifa Majid

**Affiliations:** 1 Department of Psychology, University of York, York, UK; 2 Centre for Language Studies, Radboud University, Nijmegen, The Netherlands; 3 Max Planck Institute for Psycholinguistics, Nijmegen, The Netherlands

**Keywords:** Olfaction, Odor memory, Grammatical gender, Linguistic relativity

## Abstract

Odors are often difficult to identify and name, which leaves them vulnerable to the influence of language. The present study tests the boundaries of the effect of language on odor cognition by examining the effect of grammatical gender. We presented participants with male and female fragrances paired with descriptions of masculine or feminine grammatical gender. In Experiment 1 we found that memory for fragrances was enhanced when the grammatical gender of a fragrance description matched the gender of the fragrance. In Experiment 2 we found memory for fragrances was affected by both grammatical gender and gender associations in fragrance descriptions – recognition memory for odors was higher when the gender was incongruent. In sum, we demonstrated that even subtle aspects of language can affect odor cognition.

**Electronic supplementary material:**

The online version of this article (10.3758/s13414-019-01729-0) contains supplementary material, which is available to authorized users.

## Introduction

Is the way we perceive and remember the world influenced by the language used to describe it? Although traditionally seen as separate “modules” (Fodor, 1983), there is considerable evidence that language can affect perception (for review, see Lucy, 2016). For example, words have been shown to affect perceptual discrimination of shapes (Lupyan & Spivey, 2008) and color (Winawer et al., 2007), as well as perceptual detection of visual objects (Lupyan & Ward, 2013; Ostarek & Huettig, 2017) and motion (Francken, Kok, Hagoort, & de Lange, 2015; Meteyard, Bahrami, & Vigliocco, 2007). The way objects and events are described has also been shown to affect memory for spatial configurations, motion, and path direction (Levinson, 2003; Majid, Bowerman, Kita, Haun, & Levinson, 2004) and color (Davidoff, Davies, & Roberson, 1999), for example. Although many studies support the idea that perception can be modulated by language, it remains a controversial issue (see Firestone & Scholl, 2016; Simanova, Francken, de Lange, & Bekkering, 2016). One aspect of this endeavor that may limit its progress is the overwhelming emphasis on visual perception. By expanding investigations to the less studied senses, further insights can be gleaned about how language and perception interact (Levinson & Majid, 2014; Majid et al., 2018).

One perceptual modality that may be particularly interesting to investigate from this perspective is olfaction (Speed, 2016). Although individuals may have strong opinions on whether they do or do not like an odor, our ability to imagine (Crowder & Schab, 1995) and describe odors is limited (Cain, 1979; de Wijk, Schab, & Cain, 1995). People perform poorly at naming odors correctly, even for commonly encountered “ecologically valid” odors (Cain, 1979). These olfactory limitations could be due to neurocognitive limitations in the brain (Olofsson & Gottfried, 2015), or the result of cultural experience (Majid, 2015; O’Meara & Majid, 2016). For example, speakers of some languages are just as good at naming odors as they are at naming colors (Majid & Burenhult, 2014; Majid & Kruspe, 2018). One consequence of the difficulty identifying odors (at least in the West) is that odor perception can easily be affected by contextual information (Herz, 2003, 2005). Stevenson (2011) describes a number of ways odor perception can be influenced by visual and linguistic cues – so-called “olfactory illusions.”

Olfaction, in comparison to the other perceptual modalities, may be particularly vulnerable to the influence of context because odors are not visible and they are difficult to locate and identify (Herz, 2003). In comparison to illusions in other modalities (e.g., vision), odors themselves do not typically provide a means by which the illusion can be verified. For example, in the waterfall illusion – where staring at a waterfall leads to the illusion that the rocks at the side of the waterfall are moving upwards – the illusion can be recognized based on the fact that rocks do not typically move upwards (Stevenson, 2011). When faced with ambiguous odors, in contrast, we may search for contextual information, such as language, to inform odor perception (Herz, 2000), especially because we are verbally and visually oriented. Cain (1980) describes the “unusual perceptual transformation” (Stevenson, 2011, p. 1893) one may have when learning the name of a previously unidentified odor, where a “fishy-goaty-oily” smell suddenly becomes leather (Cain, 1980, p. 352).

It has been well-documented that labels can affect the perceived pleasantness of an odor (Ayabe-Kanamura, Kikuchi, & Saito, 1997; Bensafi, Rinck, Schaal, & Rouby, 2007; de Araujo, Rolls, Velazco, Margot, & Cayeux, 2005; Djordjevic et al., 2008; Herz & Clef, 2001; Manescu, Frasnelli, Lepore, & Djordjevic, 2014). For example, Herz and Clef (2001) presented odors to participants with either a positive (e.g., *parmesan cheese*) or a negative (e.g., *vomit*) label. Participants rated odors as significantly more pleasant when they were paired with a positive compared to a negative label, even though the odors were identical. A similar study showed that the valence of an odor label (positive or negative) can modulate brain activation in the anterior cingulate cortex and medial orbitofrontal cortex, and can modulate activation in the amygdala specifically for the test odor (compared to clean air) (de Araujo et al., 2005). Labels can also affect perceived gender attributes of an odor. Zellner, McGarry, Mattern-McClory, and Abreu (2007) used labels to influence the perception of unisex fragrances: fragrances tended to be matched more with blue and green colors when described as a “male” fragrance, and with yellow, white, and pink colors when described as a “female” fragrance.

It could be argued, however, that explicitly labelling odors in such a way (e.g., “this is chest medicine” – Herz & Clef, 2001; “this is a fragrance for women” – Zellner et al., 2007) could lead participants to strategically use linguistic information to make their odor judgments (see Firestone and Scholl's (2016) third pitfall when assessing cognitive effects on perception: “Demand and response bias”). Effects of language on odor perception could therefore reflect top-down integration of explicit semantic information with an ambiguous olfactory percept. In the present study, we set out to assess a less explicit effect of language on odor cognition. We assessed the role of language on olfaction in a novel manner by manipulating the grammatical gender of descriptions of fragrances.

Grammatical gender is a system where nouns are divided into classes based on the behavior of associated linguistic elements such as articles and determiners (Corbett, 2006). Some languages (e.g., French and German) possess a grammatical gender system that is based on natural gender (i.e., masculine and feminine). However, the assignment of grammatical gender to objects is said to be semantically arbitrary: there is nothing inherently masculine or feminine about the objects to which gender is assigned, and objects often possess opposite genders across languages (e.g., “key” is masculine in German, *der Schlüssel*, but feminine in Spanish, *la llave*).

Although grammatical gender is semantically arbitrary, it has been shown to affect how people think about objects (Boroditsky, Schmidt, & Phillips, 2003; Kurinski & Sera, 2011; Phillips & Boroditsky, 2003; Sera, Berge, & Pintado, 1994). For example, Phillips and Boroditsky (2003) found that participants rated objects and people as more similar when they shared grammatical gender than when they did not. In addition to similarity, the grammatical gender of a noun can also affect the semantic associations with its referent. Boroditsky et al. (2003) asked German and Spanish speakers to list relevant adjectives for objects that had an opposite grammatical gender in each of the languages. Grammatical gender was shown to affect the types of adjectives that were provided: objects were described using more masculine adjectives when the word had masculine grammatical gender, but more feminine adjectives when the word had feminine grammatical gender. For example, the object *key* was described with adjectives such as “*hard, heavy, jagged, metal, serrated,* and *useful*” by German speakers, but as “*golden intricate, little, lovely, shiny,* and *tiny*” by Spanish speakers (although see Mickan, Schiefse, & Stefanowitsch, 2014).

Grammatical gender is an interesting linguistic feature to investigate in terms of the effect of language on odor perception because its effects are thought to be automatic and implicit. For example, effects of grammatical gender have been observed when speakers of a gendered language complete a task in English (a language with no grammatical gender system), when a non-linguistic task is used, and when engaged in verbal interference (Phillips & Boroditsky, 2003). Automatic and pre-attentive effects of grammatical gender have also been demonstrated using event-related potentials (Boutonnet, Athanasopoulos, & Thierry, 2012). This study found that grammatical gender affected a morphosyntactic marker (LAN amplitude) in Spanish-English bilinguals, but not monolingual English speakers, in a semantic categorization task of pictures. Intriguingly, the effects were restricted to event-related potentials, and not observed in behavioral measures

It should be noted, however, that other investigations have found the effects of grammatical gender to be more constrained, suggesting effects of grammatical gender may only occur under specific conditions. For example, some studies suggest that grammatical gender effects require verbalization (e.g., Kousta, Vinson, & Vigliocco, 2008; Ramos & Roberson, 2011) or use of gender-marked articles (e.g., Imai, Schalk, Saalbach, & Okada, 2014). Instructions given in a task may also be important, with some studies finding effects only when there is explicit reference to gender (e.g., Bender, Beller, & Klauer, 2016; Cubelli, Paolieri, Lotto, & Job, 2011; Ramos & Roberson, 2011; for discussion see Bender, Beller, & Klauer, 2011). It has also been suggested that effects of grammatical gender are limited to particular semantic categories, such as animate objects (e.g., Imai et al., 2014; Vigliocco, Vinson, Paganelli, & Dworzynski, 2005). Finally, effects of grammatical gender may differ across languages depending on the number of grammatical genders in the language (e.g., Koch, Zimmermann, & Garcia-Retamero, 2007; Sera, Elieff, Forbes, Burch, & Dubois, 2002; Vigliocco et al., 2005), the transparency and ubiquity of gender marking in the language (e.g., Sera et al., 2002), and whether speakers are monolingual or not (Bassetti, 2007). In the present study we used an implicit grammatical gender manipulation with nouns for inanimate objects, providing a test of some of the possible constraints on the effect of grammatical gender.

The present study advances previous work in two ways. First, we build upon research assessing the effect of language on odor cognition by using grammatical rather than explicit lexical cues to gender. Second, we assess the effect of grammatical gender on thought by asking participants to judge fragrances associated with nouns of a specific grammatical gender (i.e., depicting fragrance ingredients), rather than explicitly judging the referents of nouns (e.g., a *key*; cf. Boroditsky et al., 2003).

We presented native speakers of German and French with male and female fragrances and fragrance descriptions with nouns of masculine and feminine grammatical gender. Stimuli for both participant groups were identical, except that descriptions differed in grammatical gender, i.e., if a description was composed of masculine nouns in German, it had feminine nouns in French. Participants were not explicitly told whether the fragrances were male or female, but this was apparent in the fragrances themselves, since gender is a fundamental dimension on which they are classified (Lindqvist, 2013). After reading each description and smelling the corresponding fragrance, participants rated each fragrance on a number of dimensions. At the end of the experiment participants’ recognition memory for fragrances was tested. We predicted that the congruency between the gender of the fragrance and the grammatical gender of the nouns in the fragrance descriptions would affect how the fragrances were perceived and remembered.

## Experiment 1

### Method

#### Participants

Thirty native speakers of German (21 female, age *M*=26.9, *SD*=9.9 years) and 31 native speakers of French (20 female, age *M*=31.2, *SD* =12.8 years) participated in the experiment. We estimated that we would have sufficient power to detect effects with this sample size based on previous studies that reported effects of language on odor perception with smaller sample sizes (e.g., Herz & Clef, 2001; Herz, 2003; Zellner et al., 2007). Furthermore, the present study included at least twice as many odors as previous studies. German and French speakers were first recruited and tested in Nijmegen, the Netherlands, with additional French speakers tested in Lyon, France.

All participants were bilingual: French participants reported knowing on average 3.56 (*SD* = 1.19) languages and German speakers reported knowing on average 3.87 (*SD* = 0.90) languages (including abilities ranging from 1 “can ask directions and answer simple questions” to 5 “very fluent, can use the language as well as a native language”). Ability in a second language (the language rated with the highest ability after the native language) was rated as 3.85 (*SD* = 0.89) by French natives and 4.3 (*SD* = 0.70) by German natives. On a frequency scale of 5 (“every day”) to 1 (“hardly or not at all”), French speakers rated how often they spoke French as 4.94 (*SD* = 0.25) and how often they spoke their second language as 3.91 (*SD* = 1.17). German speakers rated how often they spoke German as 4.83 (*SD* = 0.46) and how often they spoke a second language as 4.47 (*SD* = 0.82).^1^ The most common second language for the French natives was English (62%). The most common second language for the German natives was Dutch (40%), followed by English (33%), with 23% reporting both Dutch and English as their second language.

#### Material

Four fragrances marketed for females and four marketed for males were used in the experiment. Fragrances were chosen based on online lists of fragrance bestsellers in France and Germany (see Table 1). We used a further four feminine and four masculine fragrances as distractors in the recognition test. Fragrances were sprayed onto plastic pellets and then placed inside opaque squeezy bottles.

**Table 1:** Fragrances with their marketed audiences

Fragrance	Marketing gender
Hugo Boss, Boss Orange	Female
Armani, Si	Female
Calvin Klein, Eternity	Female
Dior, J’adore	Female
Chanel, Bleu de Chanel	Male
Joop!, Homme	Male
Davidoff, Cool Water	Male
Hugo Boss, Boss Bottles	Male

Eight fragrance descriptions were created, so that each description contained three nouns that participants were told depicted fragrance ingredients. Within a description, the three nouns matched in grammatical gender; and across languages their grammatical genders were different in German versus French (see Table 2). For example, a description of one fragrance contained the ingredients pumpkin, sage, and marjoram, which are masculine nouns in German (*Kürbis, Salbei, Majoran*), but feminine nouns in French (*citrouille, sauge, marjolaine*). Nouns were presented without definite articles. Each fragrance was paired once with a grammatically female description and once with a grammatically male description, distributed across two experimental lists. Note that the fragrance descriptions were not chosen to match the true ingredients within the fragrances themselves, but instead they described plausible possible ingredients for the fragrance

**Table 2:** Fragrance descriptors for Experiment 1

German descriptors	German grammatical gender	French descriptors	French grammatical gender	English gloss
Kürbis, Salbei, Majoran	masculine	Citrouille, sauge, marjolaine	Feminine	Pumpkin, sage, marjoram
Apfel, Rhabarber, Kardamom	masculine	Pomme, rhubarbe, cardamome	Feminine	Apple, rhubarb, cardamom
Muskat, Farn, Lehm	masculine	Muscade, fougère, argile	Feminine	Nutmeg, fern, clay
Schiefer, Lavendel, Zimt	masculine	Ardoise, lavande, cannelle	Feminine	Slate, lavender, cinnamon
Zitrone, Sonnenblume, Melone	feminine	Citron, tournesol, melon	Masculine	Lemon, sunflower, melon
Iris, Ringelblume, Makrone	feminine	Iris, souci, macaron	Masculine	Iris, marigold, macaroon
Gewürznelke, Kiefer, Seife	feminine	Girofle, pin, savon	Masculine	Clove, pine, soap
Eiche, Magnolie, Zeder	feminine	Chêne, magnolia, cèdre	Masculine	Oak, magnolia, cedar

.

#### Procedure

The experiment was run using E-Prime (Version 2). Participants were informed they would read descriptions containing key ingredients of fragrances and then smell and rate fragrances. They were informed they could be tested for their memory of either descriptions or fragrances at the end of the experiment. Participants were presented with the names of the fragrance ingredients in the following sentence frame “Dieser Duft enthält…/ Ce parfum contient les éléments suivants…” (This fragrance contains notes of…). After reading each description the experimenter placed a squeezy bottle beneath the participants’ nose and squeezed three times, with a gap of around 4 s between each squeeze. After smelling each fragrance, participants completed ratings of the aroma using a visual analog scale of 0 to 100. The fragrances were rated for: (a) how likely the participant would be to buy the fragrance for their mother or sister, or (b) their father or brother; (c) how much they would be willing to pay for the fragrance (in Euros); (d) how clearly they could smell the ingredients in the fragrances; (e) how intense the fragrance was; and (f) how pleasant the fragrance was. Participants made their response by clicking on a scale. The order of fragrance presentation was randomized.

After presentation and rating of all fragrances, participants completed a fragrance recognition test. They smelled all eight fragrances again, plus eight distractor fragrances, in a random order. The fragrances were presented in squeezy bottles in the same manner as earlier. Participants were instructed to click on a box labelled “old” if they had smelled the odor earlier in the experiment or click on a box labelled “new” if they had not.

### Results

All participants and items were included in the analyses. No participants reported being aware of the grammatical gender manipulation, nor that the descriptions were not accurate reflections of the fragrance ingredients. All data were analyzed using mixed effect models in R using the lme4 package (Bates, Maechler, Bolker, & Walker, 2014) with fragrance gender (male vs. female),^2^ grammatical gender (masculine vs. feminine), language (French vs. German), and the interaction as fixed factors, and fragrance and participant as random intercepts. Models with maximal random effects did not converge in many cases. In order to keep the models as similar as possible, only random intercepts are reported. For effects of interest, when a model with a more complete random effects structure did converge, we summarize the results in a footnote. The female level of each variable was automatically coded as 0 and male as 1, and French as 0 and German as 1; and *p*-values were estimated using the lmerTest function (Kuznetsova, Brockhoff, & Christensen, 2017). Interactions were followed up by separate models with the factors of interest. We first present results from the odor recognition task in terms of total number of correctly recognized odors.^3^ We predicted that the congruence between the fragrance gender and the grammatical gender of the descriptions would affect how well the odors were remembered. We then present data from each rating scale (0–100) to explore whether the gender congruence also affected the way the odors were perceived. Results are summarized in Table 3. All analyses can be found in Supplementary Material S1

**Table 3:** Fragrance recognition accuracy (%) and mean odor ratings on a 0–100 scale for Experiment 1. Standard error of ratings are given in brackets

	French		German	
	Feminine gender	Masculine gender	Feminine gender	Masculine gender
*Fragrance recognition*				
Female fragrance	0.69 (0.06)	0.61 (0.05)	0.66 (0.06)	0.78 (0.06)
Male fragrance	0.71 (0.07)	0.86 (0.07)	0.72 (0.07)	0.77 (0.07)
*How likely are you to buy this fragrance for your mother or sister?*				
Female fragrance	38 (3.6)	37.68 (3.4)	42.29 (3.45)	35.91 (3.67)
Male fragrance	21.9 (3.98)	24.1 (4.46)	30.98 (4.53)	20.53 (4.04)
*How likely are you to buy this fragrance for your father or brother?*				
Female fragrance	15.44 (3.5)	16.28 (2.95)	14.12 (3.0)	17.28 (3.09)
Male fragrance	41.39 (5.31)	30.86 (5.01)	29.93 (5.09)	43.55 (5.4)
*How much would you pay for this fragrance?*				
Female fragrance	30.32 (3.0)	28.76 (2.74)	25.22 (2.79)	22.34 (3.05)
Male fragrance	32.42 (3.5)	27.61 (3.33)	21.85 (3.38)	25.93 (3.55)
*How clearly could you smell the ingredients in the fragrance?*				
Female fragrance	43.11 (3.6)	47.15 (3.2)	39.57 (3.25)	38.33 (3.66)
Male fragrance	40.57 (4.08)	51.11 (4.17)	51.13 (4.24)	35.93 (4.15)
*How intense is this fragrance?*				
Female fragrance	55.57 (2.74)	52.95 (2.71)	58.91 (2.75)	61.43 (2.78)
Male fragrance	59.79 (3.38)	58.13 (3.01)	64.28 (3)	64.48 (3.44)
*How pleasant is this fragrance?*				
Female fragrance	53.27 (3.21)	53.9 (2.68)	54.98 (2.72)	50.54 (3.27)
Male fragrance	62.52 (4.01)	55 (3.9)	55.68 (3.96)	53.15 (4.07)

.

#### Fragrance recognition

**Fig. 1:**
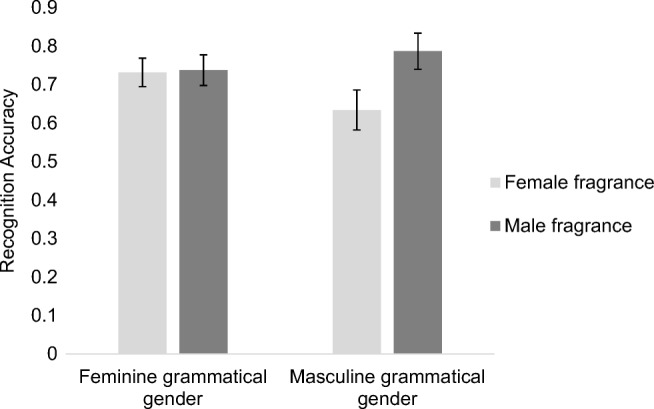
Mean fragrance recognition accuracy in Experiment 1

In line with our predictions, there was a significant interaction between fragrance gender and grammatical gender, *b* =1.26, *SE* = 0.64, *z* = 1.98, *p* = .05.^4^ Participants recognized the fragrances more accurately when the gender of the fragrance matched the grammatical gender of the description than when it did not match, as shown in Fig. 1. Follow-up models found a significant difference between male and female fragrances described with masculine nouns, *b* = 0.72, *SE* = 0.31, *z* = 2.34, *p* = .02, but no difference when described with feminine nouns *b* = -.07, *SE* = 0.32, *z* = -0.23, *p* = .82. We also assessed the effect of grammatical gender for each fragrance gender. There was no significant effect for male fragrances, *b* = 0.44, *SE* = 0.36, *z* = 1.22, *p* = .22, or female fragrances, *b* = -0.36, *SE* = 0.25, *z* = -1.4, *p* = .15, but there was a numerical trend for higher accuracy when fragrance gender and grammatical gender were congruent. There were no other significant effects.

#### Odor ratings

Below we report only significant effects within the rating data for ease. All results can be found in Supplementary Material S1.

#### How likely are you to buy this fragrance for your mother or sister?

As expected, people indicated they were more willing to buy female fragrances for their mother or sister than male fragrances, as demonstrated by a main effect of fragrance gender, *b* = -15.56, *SE* = 5.93, *t* = -2.62, *p* <.01.

#### How likely are you to buy this fragrance for your father or brother?

Conversely, participants indicated they were more likely to buy male fragrances for their father or brother than female fragrances, *b* = 23.35, *SE* = 4.43, *t* = 5.27, *p* <.001.

#### How much would you pay for this fragrance?

There were no significant effects on how much participants would be willing to pay for the fragrances.

#### How clearly could you smell the ingredients in the fragrance?

 There was a significant interaction between fragrance gender and language, *b* = 16.17, *SE* = 5.71, *t* = 2.83, *p* <.01. There was also a three-way interaction between fragrance gender, grammatical gender, and language, *b* = -21.49, *SE* = 8.15, *t* = -2.64, *p* <.01, which reflected a significant interaction between grammatical gender and language for male fragrances (*b* = -26.87, *SE* = 6.28, *t* = -4.28, *p* <.001), but not for female fragrances (*b* = -4.7, *SE* =4.84, t = .97, p = .33). Participants perceived the ingredients in male fragrances more clearly with masculine descriptions in French (effect of grammatical gender *b* = 12.39, *SE* = 4.25, *t* = 2.92, *p* =.005), but with feminine descriptions in German (effect of grammatical gender *b* = -12.69, *SE* = 4.56, *t* = -2.78, *p* =.007).^5^

#### How intense is this fragrance?

There were no significant effects in intensity ratings.

#### How pleasant is this fragrance?

There were no significant effects in pleasantness ratings.

### Discussion

We found fragrances were remembered better when they were described using nouns with grammatical gender that matched the gender of the fragrance, compared to when they did not match. Thus, Experiment 1 suggests that grammatical gender can affect the way fragrances are subsequently remembered. The effect was restricted, however, and found specifically for descriptions containing masculine nouns. This asymmetry could result from the fact that all participants in the experiment were female, making masculine attributes of the fragrances particularly salient.

In addition to the memory effect, the fragrance descriptions also affected ratings of the fragrances. Participants perceived the ingredients in male fragrances more clearly with masculine descriptions in French, but with feminine descriptions in German. This result is puzzling because it would suggest that grammatical gender behaves differently in French and German, but it could also be due to the subtleties of how grammatical gender is manifest in each of these languages. German is a three-gender language (masculine, feminine, neuter) whereas French has only two genders (masculine, feminine). It is possible that a greater number of genders within a language affects the salience of grammatical gender. Another point of difference is that the French gender system is more transparent than the German system, with phonological information being more predictive of grammatical gender (Hopp, 2013). This is supported by grammatical gender effects of language on thought in French, but not German (Sera et al., 2002). In French, masculine is the most frequently occurring grammatical form, with feminine gender marked, whereas the frequency of masculine and feminine nouns in German is similar (Hopp, 2013). This could also explain why only masculine descriptions in French affected ratings of perceived fragrance ingredients. Despite these differences, however, none satisfactorily explain the differential effects of grammatical gender in German and French.

Another possibility is that something other than grammatical gender may be driving this effect, such as gender associations. Beyond grammatical gender, people “genderize,” i.e., assign masculine and feminine attributes to objects (Yorkston & De Mello, 2005; see also Bender et al., 2016). Certain objects may be more associated with maleness and potency, and others with femaleness and beauty (Foundalis, 2002). For example, English speakers (whose language has no grammatical gender) have been shown to judge natural objects as more female, and artificial objects as more male (Forbes, Poulin-Dubois, Rivero, & Sera, 2008; Sera et al., 1994). In Experiment 1 we used nouns that may have strong gender associations. Words like *slate* and *oak* may be associated more with masculinity, whereas words like *magnolia* and *sunflower* may have more feminine associations. In Experiment 2, therefore, we re-examined the effect of grammatical gender with German speakers by orthogonally manipulating grammatical gender and gender associations.

## Experiment 2

### Method

#### Participants

Forty-two native German speakers took part in the experiment (all female, age *M* = 22.75, *SD* = 3.16 years) and were paid for their time in shopping vouchers. Due to difficulty recruiting male German participants, we decided to only recruit females since a balanced group would not be possible, and it is likely that males and females differ in their olfactory ability (for review, see Majid et al., 2017). Participants were recruited and tested in Nijmegen, the Netherlands, and Emmerich, Germany.

All participants were bilingual: this was assessed using the same scales as in Experiment 1. Participants reported knowing on average 3.83 (*SD* = 0.79) languages. Ability in a second language was rated as 4.33 (*SD* = 0.46). Participants rated how often they speak German as 4.78 (*SD* = 0.47) and how often they speak a second language as 4.25 (*SD* = 0.91). The majority of participants reported both Dutch and English as their second language (38%), with the third or more language also being either Dutch (31%) or English (31%).

#### Material

The same fragrances from Experiment 1 were used, except that because *Joop!* was rated as a female instead of a male fragrance, it was replaced with the male fragrance *Invictus*. Fragrances were presented in the same manner as Experiment 1.

Eight fragrance descriptions were used with two descriptors for each of the following four conditions: (1) masculine-association and masculine-grammatical gender, (2) masculine-association and feminine-grammatical gender, (3) feminine-association and masculine-grammatical gender, (4) feminine-association and feminine-grammatical gender. Each description contained three fragrance ingredients matching in gender association and grammatical gender (see Table 4). As a check of the manipulation of gender association, a set of 20 native English speakers (age *M* = 37.5, *SD* = 14.13 years) rated nouns for masculinity and femininity on two separate 0–100 scales. Native English speakers were used in order to rule out any influence of grammatical gender on the ratings. Ratings of masculinity were subtracted from ratings of femininity, leaving a rating of gender association on a -100 to 100 scale. Female-associated nouns (*M* = 60.61, *SE* = 4.64) were significantly more feminine than male-associated nouns (*M* = -47.47, *SE* = 3.78), *t*(22) = 18.06, *p* < .001, *d* = 7.7. Each fragrance was paired with a description in each of the four conditions, distributed across four experimental lists

**Table 4:** Fragrance descriptors from Experiment 2

English translations	German descriptors	Grammatical gender	Gender Association
Ivy, sugar, poppy	Efeu, Zucker, Mohn	Masculine	Feminine
Peach, honey, lavender	Pfirsich, Honig, Lavendel	Masculine	Feminine
Whisky, slate, chili	Whisky, Schiefer, Chili	Masculine	Masculine
Alcohol, clay, musk	Alkohol, Lehm, Moschus	Masculine	Masculine
Raspberry, vanilla, clementine	Himbeere, Vanille, Klementine	Feminine	Feminine
Strawberry, lily, rose	Erdbeere, Lilie, Rose	Feminine	Feminine
Oak, pine, ash	Eiche, Kiefer, Asche	Feminine	Masculine
Walnut, pistachio, ink	Walnuss, Pistazie, Tinte	Feminine	Masculine

.

#### Procedure

The experimental procedure was identical to that in Experiment 1.

### Results

Again, no participants reported awareness of the gender association or grammatical gender manipulation, nor that the descriptions were not accurate reflections of the fragrance ingredients. Data was analyzed in the same manner as in Experiment 1, with fragrance gender (male vs. female), grammatical gender (masculine vs. feminine), gender association (masculine vs. feminine), and the interactions as fixed factors, and fragrance and participant as random intercepts. Again, we report models with random intercepts only, since models with maximal random effects did not converge in many cases, but in cases when models of interest did converge with a more complete random effects structure, we summarize the results in a footnote. As before, we first analyzed odor recognition in terms of total number of correctly recognized odors, and then data from each judgment task. Results are summarized in Table 5

**Table 5:** Fragrance recognition accuracy (%) and mean odor ratings on a 0–100 scale for Experiment 2. Standard errors of the ratings are placed brackets

	Feminine association		Masculine association	
	Feminine gender	Masculine gender	Feminine gender	Masculine gender
*Fragrance recognition*				
Female fragrance	0.60 (0.09)	0.74 (0.11)	0.74 (0.11)	0.60 (0.09)
Male fragrance	0.57 (0.09)	0.62 (0.10)	0.71 (0.11)	0.64 (0.10)
*How likely are you to buy this fragrance for your mother or sister?*				
Female fragrance	52.08 (4.17)	49 (4.08)	32.6 (4.34)	24 (3.70)
Male fragrance	36.83 (4.39)	37.53 (4.63)	16.05 (3.29)	12.33 (3.53)
*How likely are you to buy this fragrance for your father or brother?*				
Female fragrance	3.73 (0.98)	6.5 (1.31)	21.78 (3.96)	37.6 (4.23)
Male fragrance	19.58 (4.73)	17.83 (3.45)	38.25 (4.78)	39.55 (5.19)
*How much would you pay for this fragrance?*				
Female fragrance	25.48 (2.49)	24.25 (2.66)	23.5 (2.84)	25.53 (2.85)
Male fragrance	24.98 (2.69)	24.28 (2.87)	25 (3.02)	24 (2.95)
*How clearly could you smell the ingredients in the fragrance?*				
Female fragrance	53.7 (3.98)	37.43 (4.42)	31.33 (3.26)	34.28 (3.38)
Male fragrance	42.9 (4.16)	39.95 (4.47)	30.98 (3.76)	45.2 (3.58)
*How intense is this fragrance?*				
Female fragrance	65.82 (2.29)	62 (2.58)	59.53 (2.81)	59.13 (2.50)
Male fragrance	69.1 (2.82)	66.3 (2.90)	63.9 (2.93)	69.53 (2.80)
*How pleasant is this fragrance?*				
Female fragrance	58.6 (3.68)	60.78 (3.62)	59.3 (3.52)	53.63 (3.31)
Male fragrance	58.7 (3.38)	55.85 (3.98)	55.88 (3.32)	47.95 (3.78)

.

#### Fragrance recognition

**Fig. 2:**
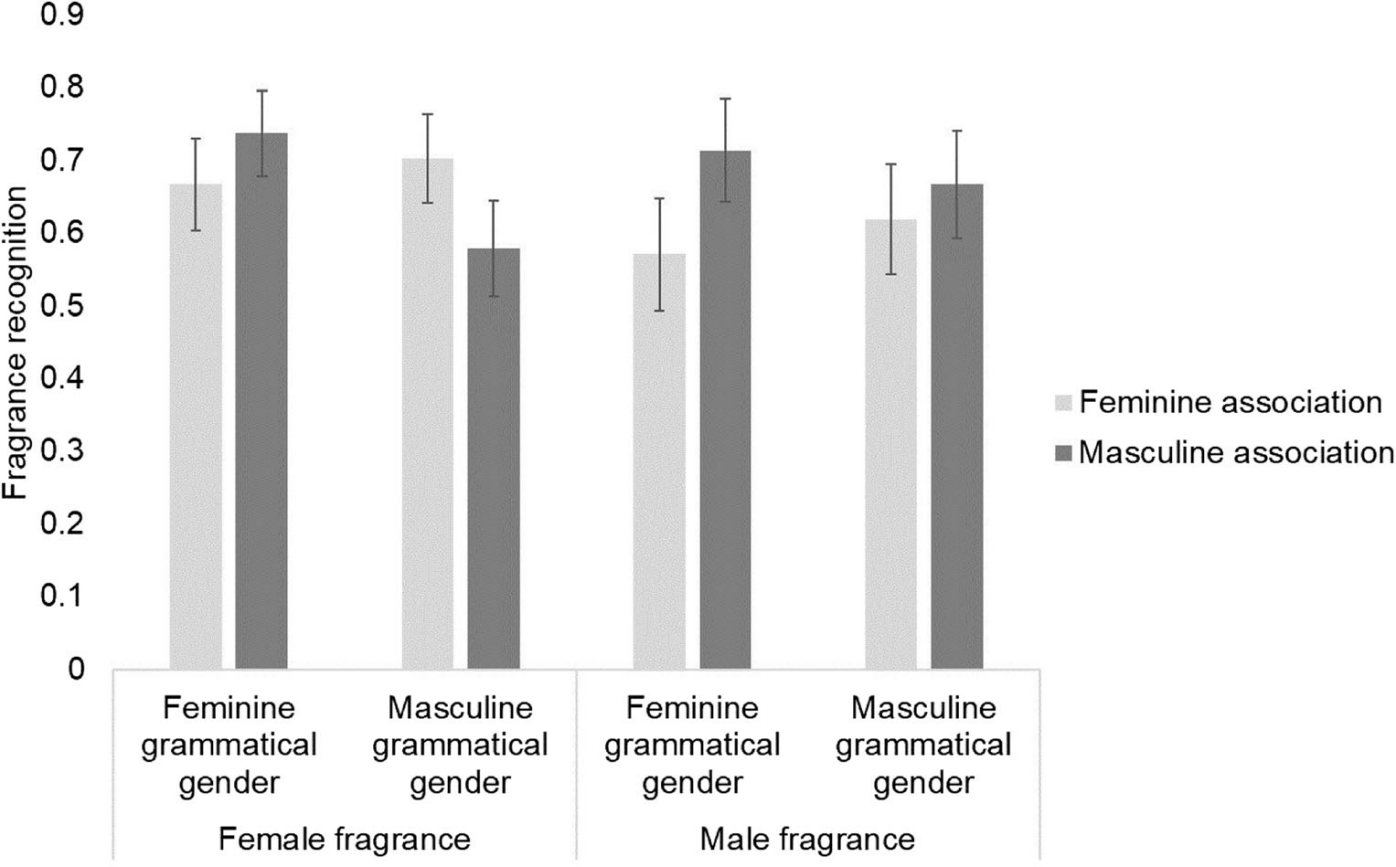
Mean fragrance recognition accuracy in Experiment 2

6For fragrance memory, grammatical gender interacted with the new factor, gender association, rather than fragrance gender, *b* = -1.30, *SE* = 0.67, *z* = -1.95, *p* = .051^7^, with recognition accuracy higher when grammatical gender and gender association mismatched compared to matched (see Fig. 2): For feminine grammatical descriptions recognition was higher with masculine association than with feminine association, *b* = 0.65, *SE* = 0.34, *z* = 1.95, *p* = .052, and although there was no significant effect of gender association for descriptions of masculine grammatical gender, *b* = -0.22, *SE* = 0.33, *z* = -.66, *p* = .51, there was a numerical trend of higher accuracy for feminine association than for masculine association. When looking separately by gender association, there was no effect of grammatical gender for masculine gender associations, *b* = -0.44, *SE* = 0.33, *z* = -1.32, *p* = .19, nor feminine gender associations, *b* = 0.43, *SE* = 0.33, *z* = 1.31, *p* = .19. This suggests both grammatical gender and gender associations affect odor memory, but that they interfere when there is matching gender.

#### How likely are you to buy this fragrance for your mother or sister?

There was a significant effect of fragrance gender, *b* = -16.74, *SE* = 5.29, *t* = -3.17, *p* = .002, where as expected, participants were more likely to buy a fragrance for their mother or sister if it was a female fragrance than if it was a male fragrance. There was also a significant effect of gender association, *b* = -19.67, *SE* = 5.29, *t* = -3.72, *p* <.001, with participants more likely to buy a fragrance for their mother or sister if it had been described using nouns with female association compared to with male association.

#### How likely are you to buy this fragrance for your father or brother?

There was a significant effect of fragrance gender, *b* = 15.87, *SE* = 6.18, *t* = 2.57, *p* = .02, where participants were more likely to buy a fragrance for their father or brother if it was a male fragrance than if it was a female fragrance. There was also a significant effect of gender association, *b* = 17.29, *SE* = 5.05, *t* = 3.43, *p* <.001, with participants more likely to buy a fragrance for their father or brother if it had been described using nouns with a male association compared to with a female association. Although the interaction between grammatical gender and gender association was not significant, *b* = 13.22, *SE* = 7.16, *t* = 1.85, *p* = .07, there was a trend showing that participants were even more likely to buy a fragrance when paired with a description with male association and male grammatical gender.

#### How much would you pay for this fragrance?

There were no significant effects on price ratings.

#### How clearly could you smell the ingredients in the fragrance?

 There was no effect of fragrance gender, *b* = -11.82, *SE* = 5.89, *t* = -2.0, *p* = .06. There was a significant effect of grammatical gender, *b* = -15.32, *SE* = 4.51, *t* = -3.4, *p* <.001, with ingredients perceived more clearly in the fragrances when they were feminine compared to masculine gender. There was also a significant effect of gender association, *b* = -20.84, *SE* = 4.51, *t* = -4.62, *p* <.001, with ingredients perceived more clearly when they had a female compared to a male association. It is possible that ratings were higher for the feminine compared to the masculine levels of the three variables because all participants were female. Higher ratings may reflect more familiarity or affiliation with the female associations.

**Fig. 3:**
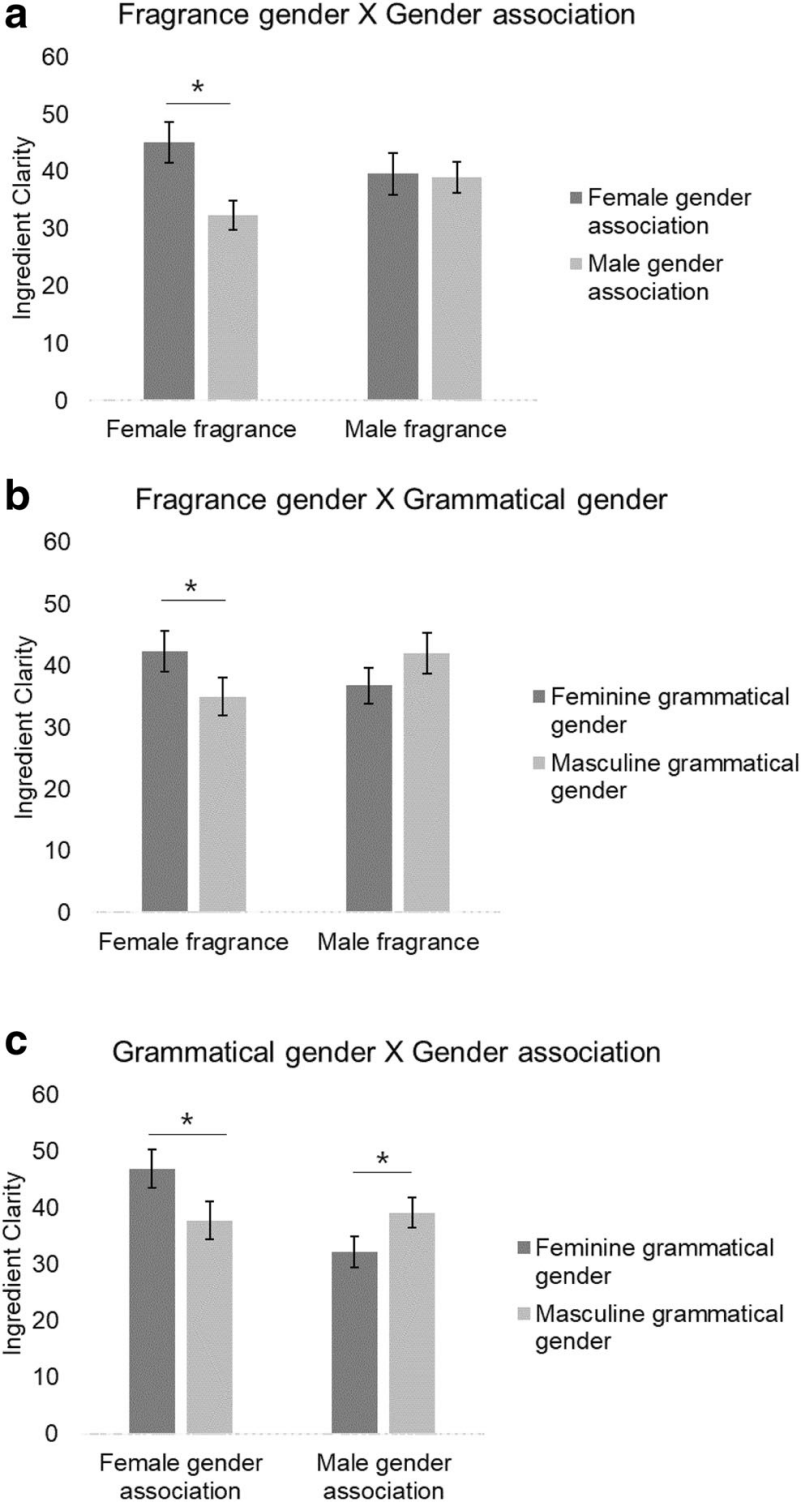
Mean ratings of ingredient clarity in Experiment 2 for (**a**) fragrance gender by grammatical gender interaction, (**b**) fragrance gender by gender association interaction, and (**c**) gender association by grammatical gender interaction

We observed a significant interaction between fragrance gender and gender association, *b* = 12.50, *SE* = 6.37, *t* = 1.96, *p* = .05. For female fragrances, ratings were higher with feminine than with masculine association, *b* = -21.79, *SE* = 3.01, *t* = -4.25, *p* <.001, whereas there was no difference in ratings between masculine and feminine association for male fragrances, *b* = -1.24, *SE* = 3.40, *t* = -0.36, *p* = .72. There was also a significant interaction between fragrance gender and grammatical gender, *b* = 13.44, *SE* = 6.40, *t* = 2.10, *p* = .04. Ingredients were perceived more clearly when grammatical gender and fragrance gender matched compared to when they mismatched: Ratings were significantly higher for female fragrances when paired with a description of feminine grammatical gender compared to masculine grammatical gender, *b* = -7.2, *SE* = 3.17, *t* = -2.28, *p* = .02, and although the difference between masculine and feminine gender for male fragrances was not significant, *b* = 5.62, *SE* = 3.36, *t* = 1.67, *p* = .10, there was a numerical trend for ratings to be higher for masculine than feminine gender. We also observed an interaction between grammatical gender and gender association, *b* = 16.46, *SE* = 6.40, *t* = 2.57, *p* = .01. Ingredients were perceived more clearly when grammatical gender and gender association matched compared to when they mismatched: Ratings were significantly higher for fragrances with descriptions of feminine gender associations and feminine grammatical gender compared to male grammatical gender, *b* = -8.78, *SE* = 3.20, *t* = -2.74, *p* = .007, and ratings were significantly higher for fragrances with descriptions of masculine gender associations and masculine grammatical gender compared to feminine grammatical gender *b* = 7.42, *SE* = 3.16, *t* = 2.34, *p* = .02. Interactions are depicted in Fig. 3.^8^

#### How intense is this fragrance?

There were no significant effects in ratings of intensity.

#### How pleasant is this fragrance?

There were no significant effects in ratings of pleasantness.

### Discussion

Experiment 2 also demonstrated an effect of linguistic descriptions on olfactory memory. But in contrast to Experiment 1, the effect of the description did not depend on the gender of the fragrance. In addition, there was no longer a benefit in memory for congruence. Instead, higher recognition scores occurred when grammatical gender and gender association were incongruent. This effect was asymmetrical: for descriptions with feminine grammatical gender there was a significant difference between those with masculine versus feminine gender association; but this was not the case for descriptions with masculine grammatical gender (although the same pattern held). This asymmetry was opposite to that in Experiment 1, where a significant difference was only found for descriptions with masculine grammatical gender. Since different ingredient nouns were used across the two experiments, we cannot rule out that these differences are due to specific items. This would mean that the strength of association between words and their grammatical gender may vary across items.

As in Experiment 1, the gender of the fragrance was a significant factor in the likelihood that participants would buy a fragrance for their mother or sister, or their father or brother. In addition, in Experiment 2 we found that gender association played a similar role. When a fragrance was described with nouns with a feminine association, participants indicated they were more likely to buy that fragrance for their mother or sister than when it was described with nouns with a masculine association (and vice versa for masculine association and the likelihood of buying for their father or brother). This demonstrates that the gender association manipulation successfully elicited the intended masculine and feminine associations.

Manipulations of gender association, grammatical gender, and fragrance gender also affected ratings of how well the ingredients could be perceived in the fragrances. The overall pattern suggests that across the three variables, congruence in gender led to higher ratings of ingredient clarity (see Fig. 3). It is likely that participants used this congruence as a positive cue when comparing the descriptions with the actual fragrances. By orthogonally manipulating grammatical gender and gender association, we have shown the individual effect of each. This supports the decision following Experiment 1 to include gender association in the experimental design.

## General discussion

Using a manipulation of grammatical gender, we show for the first time that odor cognition can be implicitly affected by language, i.e., participants were not consciously aware of the semantic information conveyed by grammatical gender but nevertheless their olfactory memory was affected. We further demonstrated an implicit effect of language by using nouns with masculine or feminine associations. In comparison to previous studies investigating the semantic effects of grammatical gender, our results are more difficult to explain in terms of explicit or strategic use of grammatical gender during the task (cf. Bender et al., 2016). Judgments were not explicitly made on the referents of the gendered nouns, but instead on fragrances associated with nouns. Moreover, the combination of the odors of three objects in a fragrance were considered at once. We note, however, that with our current data we cannot determine whether the linguistic effect of gender on memory was due to congruence during perceptual encoding or at a later decision stage (cf. Mitterer, Horschig, Müsseler, & Majid, 2009).

We note that some caution may be necessary with regard to the generalizability of our findings, due to constraints of the linear mixed effects models used. We report models with random effects of participants and fragrances modelled as intercepts only. Multiple models used in the present analyses did not converge when random slopes were entered, and so, for the sake of parsimony, we report models with random intercepts only. However, these models have been shown to generalize less well than models that also include random slopes (Barr, Levy, Scheepers, & Tily, 2013).

In Experiment 1, we found that memory for fragrances was more accurate when the fragrance description contained nouns with grammatical gender that matched the gender of the fragrance. This is in line with previous studies that have shown facilitated sentence comprehension for gender congruent information, in comparison to gender incongruent and neutral information (Friederici & Jacobsen, 1999; Guillelmon & Grosjean, 2001). In the present study, it is possible that grammatical gender in the fragrance descriptions activated gender information, which then facilitated encoding of fragrances that agreed with this gender (comparable to gender priming effects in sentence processing, Friederici & Jacobsen, 1999). In comparison, in Experiment 2, we found that memory for fragrances was more accurate when the fragrance description was incongruent in terms of grammatical gender and gender association. However, we approach this result with caution since the effect was not found in a statistical model with a more complete random effects structure that tested the collapsed variable “congruence.” It is possible then that this finding is in line with Bender et al. (2016), who found that effects of grammatical gender disappeared when grammatical gender and gender associations were orthogonal. If fragrance memory was indeed more accurate when grammatical gender and gender association were congruent, then what could explain the contrast with the results of Experiment 1?

We see two possible explanations for the difference. First, in Experiment 2, grammatical gender and gender associations came from the same source at the same time (i.e., the words). In comparison, in Experiment 1, the words contained only one form of gender information: grammatical gender. It could be argued then that two pieces of gender information in the same source leads to interference. The second possibility is that when multiple pieces of gender information associated with a word are congruent, they become redundant or they assimilate into one piece of gender information. In contrast, when grammatical gender and gender associations are incongruent, the two cannot assimilate, leaving two separate pieces of gender information that need to be reconciled. Having a greater number of attributes associated with an odor has been shown to lead to a stronger memory trace (Lyman & McDaniel, 1990). For example, Lyman and McDaniel (1990) found memory for odors was higher when an odor was paired with an odor name and a picture, compared to conditions when the odor was paired with only an odor name or only a picture. Therefore, it is possible that the fragrances in Experiment 2 were remembered better in the incongruent condition because they were paired with grammatical gender, and also a gender association.

In Experiment 2 we also found that the congruence between grammatical gender, gender association, and fragrance gender affected judgments of how clearly the participants could perceive the ingredients in the fragrance. As a reminder, the fragrances did not in fact match the ingredients in the fragrance descriptions, so this suggests language plays a powerful role in shaping olfactory percepts. Participants used the gender information implicit in the descriptions and the fragrance to make their judgments. Here, in comparison to the memory effect, we found a positive effect of congruence, with higher ratings of ingredient clarity when gender was congruent. It is possible that gender congruence initially evoked positive responses, but when encoded in memory, the congruence assimilated into a single piece of information, leading to a memory advantage for incongruent gender information.

We used fragrances in the present study, which contain a variety of different scents, thereby making it difficult to perceive all individual ingredients (Laing & Francis, 1989). It is possible, therefore, that we have tested an effect of language when odor cognition is most fragile. Research in other perceptual modalities suggests that language is more likely to affect perception when perception is difficult (Ma, Zhou, Ross, Foxe, & Parra, 2009; Pavan, Skujevskis, & Baggio, 2013) or uncertain (Cibelli, Xu, Austerweil, Griffiths, & Regier, 2016), or when the domain is abstract (e.g., time; Boroditsky et al., 2003). Similarly, Herz and Clef (2001) used odors deliberately chosen for their ambiguity – odors that could have at least two possible sources. Further research is required to assess whether effects of grammatical gender and gender associations would similarly be observed for odors that are less complex or more familiar.

It is interesting to note that no effects of descriptions on ratings of pleasantness or intensity were found. Many previous studies have demonstrated the effect of labels on odor pleasantness, including the effect of positive versus negative labels (Ayabe-Kanamura, Kikuchi, & Saito, 1997; Bensafi et al., 2014, 2007; de Araujo et al., 2005; Djordjevic et al., 2008; Laudien, Wencker, Ferstl, & Pause, 2008; Manescu et al., 2014), as well as the presence versus absence of an odor name (Distel & Hudson, 2001; Ferdenzi et al., 2016). Similarly, effects of labels on intensity have been observed (Distel & Hudson, 2001; Manescu et al., 2014). One possibility for why we did not observe effects in pleasantness or intensity ratings is because our linguistic manipulation was fairly implicit, whereas in previous studies the presentation of labels has been explicit. This is in line with the proposal by Speed and Majid (2018) that language affects odor perception at a high-level lexical semantic stage, rather than in lower level perceptual processes. Another possible explanation is that gender information is irrelevant for the relationship between odor valence, odor intensity, and odor identification (Distel et al., 1999; Distel & Hudson, 2001). Importantly, though, gender in language affects memory for odors without awareness of the gender information or the need for explicit memorizing of odors and labels.

The finding that grammatical gender affected our implicit measure (i.e., memory) and not explicit ratings such as “*how pleasant is this fragrance?*” is in contrast to previous studies suggesting effects of grammatical gender are only observed with explicit tasks (e.g., Bender et al., 2011, 2016; Cubelli et al., 2011; Ramos & Roberson, 2011). This suggests that grammatical gender can affect cognition more implicitly than previously thought. This may be particularly likely for odor memory, because odors are difficult to conceptualize (Cain, 1979; de Wijk, Schab, & Cain, 1995), and odor perception is easily influenced by language (Herz, 2003). The fragrance descriptions may therefore have been the most reliable information with which to encode the odors in memory. On the other hand, the explicit judgments of odors may have relied more on other salient factors such as perceived pleasantness and personal preference, which is thought to be an early component of odor perception (Khan et al., 2007; Majid, Burenhult, Stensmyr, de Valk, & Hansson, 2018; Yeshurun & Sobel, 2010). In comparison, for tasks requiring judgments of the gender or semantic similarity of objects as words or pictures (e.g., Bender et al., 2011; Cubelli et al., 2011; Ramos & Roberson, 2011), grammatical gender information may be the most salient information to use in the judgment. Note that when the present participants were asked to consider both the fragrances and the descriptions (i.e., “how clearly can you perceive the ingredients in the fragrance?”), grammatical gender, gender association, and fragrance gender interacted. This could be considered more comparable to explicit gender or semantic similarity judgments.

There are some methodological considerations that should be kept in mind when interpreting the present results. Firstly, each noun’s grammatical gender was determined based on a dictionary entry. However, it could be better for future studies to ask participants to explicitly state the grammatical gender of each noun after the experiment to better capture individual speaker language use. In a similar vein, the linguistic background of participants could be better incorporated into future study design. Many of the German participants identified Dutch, another gendered language, as their second language. It is possible that any incongruence between German grammatical gender and Dutch grammatical gender could have reduced effects seen here (cf. studies showing between-language gender competition effects; Hopp & Lemmerth, 2016; Morales, Paolieri, Dussias, Kroff, Gerfen, & Bajo, 2016; Sabourin & Stowe, 2008; Weber & Paris, 2004).

The effect of grammatical gender also interacted with gender associations (Experiment 2). A potential limitation to this finding, however, is that ratings of gender associations were collected from native English speakers, and not native speakers of German. We judged this to be the most appropriate way to gauge gender associations of nouns so as to avoid contamination of grammatical gender. It is possible, however, that such gender associations differ culturally (cf. Beller, Brattebø, Lavik, Reigstad, & Bender, 2015). For example, it is common in English to refer to boats as females (Nicoladis & Foursha-Stevenson, 2012). The gender ratings could therefore reflect specific cultural notions rather than “universal” gender associations per se, and as such differ from gender associations in German.

It must also be acknowledged that the majority of participants in Experiment 1 were female, and all participants in Experiment 2 were female. This could have implications for the present results in two ways. Firstly, many studies have suggested there are differences in olfactory perception and identification between males and females (Majid et al., 2017), which could lead to gender differences in susceptibility to linguistic influence on olfaction. Secondly, a noun’s gender associations could be more or less salient to a participant depending on how congruent they are with their own gender. However, the difference in fragrance recognition for fragrances paired with masculine and feminine nouns in Experiment 2 was comparable for nouns with male and female associations, suggesting both male and female associations were salient.

## Conclusion

Even though descriptions about fragrance families and notes are available to help individuals choose a fragrance, consumers are thought to have difficulty using this information to aid in their decision making, and instead: “marketers default to images of beautiful people, and the sales clerks to the reassurance that a bottle is ‘new,’ ‘popular,’ or ‘my favorite’ ” (Donna, 2009, p. 27). The present results suggest that the language used to describe fragrances could, in fact, be a powerful influence on their success.

We demonstrate that odor cognition is sensitive to manipulations of grammatical gender and gender associations. Thus, we can manipulate the way that odors are remembered in a subtle, non-explicit manner. At the same time, this study shows that the effect of grammatical gender can go beyond judgments of individual objects to their odors, as perceived in complex fragrances. Gender is a pervasive feature of some languages, both in grammar and in terms of associations with gender. Although consumers may feel lost in the language of fragrance, language is indeed a powerful tool in shaping fragrance perception and affecting the likelihood that a fragrance is remembered.

## Electronic supplementary material

ESM 1 (DOCX 35 kb)

## Footnotes

1 When more than one second language was identified, frequency ratings for the most frequently used language were used. The same criterion was applied in Experiment 2.

2 A separate set of 33 participants smelled each fragrance and rated to what extent they thought it was for a man or a woman. Based on these ratings, Joop! was categorized as a female fragrance instead of a male fragrance.

3 Note that because our design was not blocked, we cannot analyze other measures of recognition such as sensitivity (*d’*).

4 The model did not converge with random intercepts for both participants and fragrances, so here we report the model with only random intercepts by participants. Note than in a model with participants as random intercepts and slopes, we also find an effect of congruency (collapsing across grammatical gender and fragrance gender), *b* = -0.44, *SE* = 0.22, *z* = -1.96, *p* = .04.

5 Note that in a model with a complete random effects structure, with random intercepts and slopes for participants and fragrances, the pattern of results remained the same: interaction between fragrance gender and language *b* = 16.23, *SE* = 5.78, *t* = 2.81, *p* <.01, interaction between fragrance gender, grammatical gender, and language *b* = -21.49, *SE* = 7.92, *t* = -2.72, *p* <.01

6 The model did not converge with random intercepts for participants and fragrances, so here we report the model with only random intercepts by participants.

7 We note that in a model with a more complete random effects structure including participants modelled as random intercepts, slopes, and fragrance as random intercept, and with congruency collapsed across gender, the effect of congruence between grammatical gender association was not significant, *b* = 0.43, *SE* = 0.26, *t* = 1.65, *p* = .10.

8 Note that in a model with a more complete random effects structure including random intercepts and slopes for participants, and a random intercept only for fragrance, the pattern of results remained the same: interaction between fragrance gender and grammatical gender *b* = 13.38, *SE* = 6.16, *t* = 2.17, *p* =.03, interaction between fragrance gender and gender association *b* = 12.49, *SE* = 6.13, *t* = 2.04, *p* = .04, interaction between grammatical gender and gender association, *b* = 16.27, *SE* = 6.16, *t* = 2.64, *p* = .01.
