# Supplementary material for: Linguistic features of fragrances: The role of grammatical gender and gender associations
Source: Atten Percept Psychophys. 2019 May 1;81(6):2063–77. doi: 10.3758/s13414-019-01729-0 (PMC6675776; doi:10.3758/s13414-019-01729-0)
Supplement: Supplementary file 1 — (DOCX 35 kb) [file 13414_2019_1729_MOESM1_ESM.docx]

**S1. Experiment 1 Summary of mixed effects models**

**S1.1. Fragrance Recognition**

*Data.lmer = glmer(Accuracy ~ FragranceGender*GrammaticalGender*Language + (1|Subject), family=binomial, Data)*

Random effects

| **Group** | **Name** | **Var** | ***SD*** |
| --- | --- | --- | --- |
| Subject | Intercept | 0.15 | 0.39 |

| **Variable** | **Coeff. Est.** | ***SE*** | ***z*** | ***p*** |
| --- | --- | --- | --- | --- |
| Intercept | 0.78 | 0.26 | 3.03 | .002 |
| Fragrance Gender | 0.03 | 0.41 | 0.07 | .94 |
| Grammatical Gender | -0.26 | 0.34 | -0.77 | .44 |
| Language | 0.49 | 0.39 | 1.26 | .21 |
| Fragrance Gender*Grammatical Gender | 1.26 | 0.64 | 1.98 | .05 |
| Fragrance Gender*Language | -0.26 | 0.60 | -0.43 | .67 |
| Grammatical Gender*Language | -0.23 | 0.51 | -0.44 | .66 |
| Fragrance Gender*Grammatical Gender*Language | -.76 | 0.88 | -0.86 | .39 |

Fixed effects

**Follow-up tests**

**S1.1.1. Grammatical gender = masculine**

*Data.lmer = glmer(Accuracy ~ FragranceGender + (1|Subject), family=binomial, Data)*

Random effects

| **Group** | **Var** | ***SD*** |
| --- | --- | --- |
| Subject | 0 | 0 |

Fixed effects

| **Variable** | **Coeff. Est.** | ***SE*** | ***z*** | ***p*** |
| --- | --- | --- | --- | --- |
| Intercept | 0.62 | 0.17 | 3.67 | < .001 |
| Fragrance Gender | 0.72 | 0.31 | 2.34 | .02 |

**S1.1.2. Grammatical gender = feminine**

*Data.lmer = glmer(Accuracy ~ FragranceGender + (1|Subject), family=binomial, Data)*

Random effects

| **Group** | **Name** | **Var** | ***SD*** |
| --- | --- | --- | --- |
| Subject | Intercept | 0.70 | 0.84 |

Fixed effects

| **Variable** | **Coeff. Est.** | ***SE*** | ***z*** | ***p*** |
| --- | --- | --- | --- | --- |
| Intercept | 1.12 | 0.24 | 4.71 | < .001 |
| Fragrance Gender | -0.07 | 0.32 | -0.23 | .82 |

**S1.1.3. Fragrance gender = male**

*Data.lmer = glmer(Accuracy ~ GrammaticalGender + (1|Subject), family=binomial, Data)*

Random effects

| **Group** | **Name** | **Var** | ***SD*** |
| --- | --- | --- | --- |
| Subject | Intercept | 0.41 | 0.64 |

Fixed effects

| **Variable** | **Coeff. Est.** | ***SE*** | ***z*** | ***p*** |
| --- | --- | --- | --- | --- |
| Intercept | 1.01 | 0.28 | 3.54 | < .001 |
| Grammatical Gender | 0.44 | 0.36 | 1.22 | .22 |

**S1.1.4. Fragrance gender = female**

*Data.lmer = glmer(Accuracy ~ GrammaticalGender + (1|Subject), family=binomial, Data)*

Random effects

| **Group** | **Name** | **Var** | ***SD*** |
| --- | --- | --- | --- |
| Subject | Intercept | 0.08 | 0.29 |

Fixed effects

| **Variable** | **Coeff. Est.** | ***SE*** | ***z*** | ***p*** |
| --- | --- | --- | --- | --- |
| Intercept | 1.00 | 0.19 | 5.12 | < .001 |
| Grammatical Gender | -0.36 | 0.25 | -1.43 | .15 |

**S1.2. How likely are you to buy this fragrance for your mother or sister?**

*RT.lmer = lmer(VasScore ~ FragranceGender*GrammaticalGender*Language + (1|Fragrance) + (1|Subject), Data)*

Random effects

| **Group** | **Name** | **Var** | ***SD*** |
| --- | --- | --- | --- |
| Subject | Intercept | 118.14 | 10.87 |
| Fragrance | Intercept | 21.05 | 4.59 |

| **Variable** | **Coeff. Est.** | ***SE*** | ***t*** | ***p*** |
| --- | --- | --- | --- | --- |
| Intercept | 38.84 | 4.08 | 9.51 | < .001 |
| Fragrance Gender | -15.56 | 5.93 | -2.62 | .01 |
| Grammatical Gender | -1.63 | 4.19 | -0.39 | .70 |
| Language | 2.80 | 5.04 | 0.55 | .58 |
| Fragrance Gender*Grammatical Gender | 2.69 | 6.95 | 0.39 | .70 |
| Fragrance Gender*Language | 6.23 | 6.95 | 0.90 | .37 |
| Grammatical Gender*Language | -3.84 | 5.98 | -0.64 | .52 |
| Fragrance Gender*Grammatical Gender*Language | -7.62 | 9.99 | -0.77 | .44 |

Fixed effects

**S1.3. How likely are you to buy this fragrance for your father or brother?**

*RT.lmer = lmer(VasScore ~ FragranceGender*GrammaticalGender*Language + (1|Fragrance) + (1|Subject), Data)*

| **Group** | **Name** | **Var** | ***SD*** |
| --- | --- | --- | --- |
| Subject | Intercept | 144.44 | 12.02 |
| Fragrance | Intercept | 1.41 | 1.19 |

Random effects

Fixed effects

| **Variable** | **Coeff. Est.** | ***SE*** | ***t*** | ***p*** |
| --- | --- | --- | --- | --- |
| Intercept | 14.63 | 3.43 | 4.27 | < .001 |
| Fragrance Gender | 23.35 | 4.43 | 5.27 | < .001 |
| Grammatical Gender | 2.38 | 3.72 | 0.64 | .52 |
| Language | 0.40 | 4.84 | 0.08 | .94 |
| Fragrance Gender*Grammatical Gender | -7.78 | 6.19 | -1.26 | .21 |
| Fragrance Gender*Language | -7.28 | 6.17 | -1.18 | .24 |
| Grammatical Gender*Language | -0.27 | 5.31 | -0.05 | .96 |
| Fragrance Gender*Grammatical Gender*Language | 14.68 | 8.81 | 1.67 | .10 |

**S1.4. How much would you pay for this fragrance?**

*RT.lmer = lmer(VasScore ~ FragranceGender*GrammaticalGender*Language + (1|Fragrance) + (1|Subject), Data)*

Random effects

| **Group** | **Name** | **Var** | ***SD*** |
| --- | --- | --- | --- |
| Subject | Intercept | 183.27 | 13.54 |
| Fragrance | Intercept | 7.79 | 2.74 |

| **Variable** | **Coeff. Est.** | ***SE*** | ***t*** | ***p*** |
| --- | --- | --- | --- | --- |
| Intercept | 30.24 | 3.19 | 9.47 | < .001 |
| Fragrance Gender | 0.40 | 3.43 | 0.12 | .91 |
| Grammatical Gender | -1.39 | 2.38 | -0.58 | .56 |
| Language | -4.75 | 4.21 | -1.12 | .26 |
| Fragrance Gender*Grammatical Gender | -1.20 | 3.98 | -0.30 | .76 |
| Fragrance Gender*Language | -2.84 | 3.96 | -0.72 | .47 |
| Grammatical Gender*Language | -1.99 | 3.40 | -0.59 | .56 |
| Fragrance Gender*Grammatical Gender*Language | 7.26 | 5.66 | 1.28 | .20 |

*Fixed effects*

**S1.5. How clearly could you smell the ingredients in the fragrance?**

*RT.lmer = lmer(VasScore ~ FragranceGender*GrammaticalGender*Language + (1|Fragrance) + (1|Subject), Data)*

Random effects

| **Group** | **Name** | **Var** | ***SD*** |
| --- | --- | --- | --- |
| Subject | Intercept | 170.67 | 13.06 |
| Fragrance | Intercept | 14.58 | 3.82 |

Fixed effects

| **Variable** | **Coeff. Est.** | ***SE*** | ***t*** | ***p*** |
| --- | --- | --- | --- | --- |
| Intercept | 43.53 | 3.77 | 11.54 | < .001 |
| Fragrance Gender | -5.92 | 4.89 | -1.21 | .24 |
| Grammatical Gender | 4.54 | 3.44 | 1.32 | .19 |
| Language | -4.32 | 4.80 | -0.90 | .37 |
| Fragrance Gender*Grammatical Gender | 9.84 | 5.73 | 1.72 | .09 |
| Fragrance Gender*Language | 16.17 | 5.71 | 2.83 | .004 |
| Grammatical Gender*Language | -4.75 | 4.91 | -0.97 | .33 |
| Fragrance Gender*Grammatical Gender*Language | -21.49 | 8.15 | -2.64 | .01 |

**Follow-up tests**

**S1.5.1. Fragrance gender = male**

*RT.lmer = lmer(VasScore ~ GrammaticalGender*Language + (1|Fragrance) + (1|Subject), Data)*

Random effects

| **Group** | **Name** | **Var** | ***SD*** |
| --- | --- | --- | --- |
| Subject | Intercept | 198.43 | 14.09 |
| Fragrance | Intercept | 2.43 | 1.56 |

Fixed effects

| **Variable** | **Coeff. Est.** | ***SE*** | ***t*** | ***p*** |
| --- | --- | --- | --- | --- |
| Intercept | 38.23 | 4.11 | 9.31 | < .001 |
| Grammatical Gender | 13.26 | 4.41 | 3.01 | .003 |
| Language | 12.11 | 5.68 | 2.13 | .04 |
| Grammatical Gender*Language | -26.87 | 6.28 | -4.28 | < .001 |

**S1.5.2. Fragrance gender = female**

*RT.lmer = lmer(VasScore ~ GrammaticalGender*Language + (1|Fragrance) + (1|Subject), Data)*

Random effects

| **Group** | **Name** | **Var** | ***SD*** |
| --- | --- | --- | --- |
| Subject | Intercept | 189.01 | 4.58 |
| Fragrance | Intercept | 20.96 | 20.82 |

Fixed effects

| **Variable** | **Coeff. Est.** | ***SE*** | ***t*** | ***p*** |
| --- | --- | --- | --- | --- |
| Intercept | 43.93 | 3.99 | 11.01 | < .001 |
| Grammatical Gender | 3.75 | 3.39 | 1.10 | .27 |
| Language | -4.34 | 4.89 | -0.89 | .38 |
| Grammatical Gender*Language | -4.7 | 4.84 | -0.97 | .33 |

**S1.5.3. Fragrance gender = male, Language = French**

*RT.lmer = lmer(VasScore ~ GrammaticalGender + (1|Fragrance) + (1|Subject), Data)*

Random effects

| **Group** | **Name** | **Var** | ***SD*** |
| --- | --- | --- | --- |
| Subject | Intercept | 322.49 | 17.96 |
| Fragrance | Intercept | 1.96 | 1.40 |

| **Variable** | **Coeff. Est.** | ***SE*** | ***t*** | ***p*** |
| --- | --- | --- | --- | --- |
| Intercept | 38.67 | 4.46 | 8.66 | < .001 |
| Grammatical Gender | 12.39 | 4.25 | 2.92 | .005 |

Fixed effects

**S1.5.4. Fragrance Gender = male, Language = German**

*RT.lmer = lmer(VasScore ~ GrammaticalGender + (1|Fragrance) + (1|Subject), Data)*

Random effects

| **Group** | **Name** | **Var** | ***SD*** |
| --- | --- | --- | --- |
| Subject | Intercept | 73.37 | 8.57 |
| Fragrance | Intercept | 7.99 | 2.83 |

Fixed effects

| **Variable** | **Coeff. Est.** | ***SE*** | ***t*** | ***p*** |
| --- | --- | --- | --- | --- |
| Intercept | 49.88 | 3.92 | 12.74 | < .001 |
| Grammatical Gender | -12.69 | 4.56 | -2.78 | .007 |

**S1.6. How intense is this fragrance?**

*RT.lmer = lmer(VasScore ~ FragranceGender*GrammaticalGender*Language + (1|Fragrance) + (1|Subject), Data)*

Random effects

| **Group** | **Name** | **Var** | ***SD*** |
| --- | --- | --- | --- |
| Subject | Intercept | 113.09 | 6.82 |
| Fragrance | Intercept | 46.52 | 16.26 |

Fixed effects

| **Variable** | **Coeff. Est.** | ***SE*** | ***t*** | ***p*** |
| --- | --- | --- | --- | --- |
| Intercept | 54.78 | 4.05 | 13.52 | < .001 |
| Fragrance Gender | 4.60 | 5.86 | 0.79 | .45 |
| Grammatical Gender | -0.80 | 2.64 | -0.30 | .76 |
| Language | 4.93 | 3.80 | 1.30 | .20 |
| Fragrance Gender*Grammatical Gender | -1.60 | 4.41 | -0.36 | .72 |
| Fragrance Gender*Language | -2.15 | 4.37 | -0.49 | .62 |
| Grammatical Gender*Language | 1.08 | 3.76 | 0.29 | .76 |
| Fragrance Gender*Grammatical Gender*Language | 4.93 | 6.26 | 0.79 | .43 |

**S1.7. How pleasant is this fragrance?**

*RT.lmer = lmer(VasScore ~ FragranceGender*GrammaticalGender*Language + (1|Fragrance) + (1|Subject), Data)*

Random effects

| **Group** | **Name** | **Var** | ***SD*** |
| --- | --- | --- | --- |
| Subject | Intercept | 120.31 | 2.21 |
| Fragrance | Intercept | 4.86 | 21.62 |

Fixed effects

| **Variable** | **Coeff. Est.** | ***SE*** | ***t*** | ***p*** |
| --- | --- | --- | --- | --- |
| Intercept | 54.01 | 3.12 | 17.33 | < .001 |
| Fragrance Gender | 9.55 | 4.89 | 1.95 | .06 |
| Grammatical Gender | -0.07 | 3.17 | -0.02 | .98 |
| Language | 0.27 | 4.26 | 0.06 | .95 |
| Fragrance Gender*Grammatical Gender | -5.63 | 6.39 | -0.88 | .38 |
| Fragrance Gender*Language | -3.86 | 6.44 | -0.60 | .55 |
| Grammatical Gender*Language | -3.74 | 4.52 | -0.83 | .41 |
| Fragrance Gender*Grammatical Gender*Language | 4.60 | 9.07 | 0.51 | .61 |

**S2. Experiment 2 Summary of mixed effects models**

**S2.1. Fragrance Recognition**

*Data.lmer = glmer(Accuracy ~ FragranceGender*GrammaticalGender*GenderAssociation + (1|Fragrance) + (1|Subject), Data, family=binomial)*

Random effects

| **Group** | **Name** | **Var** | ***SD*** |
| --- | --- | --- | --- |
| Subject | Intercept | 0 | 0 |

Fixed effects

| **Variable** | **Coeff. Est.** | ***SE*** | ***z*** | ***p*** |
| --- | --- | --- | --- | --- |
| Intercept | 0.39 | 0.31 | 1.23 | 22 |
| Fragrance Gender | -0.10 | 0.44 | -0.22 | .82 |
| Grammatical Gender | 0.65 | 0.47 | 1.38 | .17 |
| Gender Association | 0.65 | 0.47 | 1.38 | .17 |
| Fragrance Gender*Grammatical Gender | -0.45 | 0.65 | -0.70 | .49 |
| Fragrance Gender* Gender Association | -0.02 | 0.66 | -0.03 | .97 |
| Grammatical Gender* Gender Association | -1.30 | 0.67 | -1.95 | .05 |
| Fragrance Gender*Grammatical Gender*Gender Association | 0.88 | 0.93 | 0.95 | .34 |

**Follow-up tests**

**S2.1.1. Grammatical gender = feminine**

*Data.lmer = glmer(Accuracy ~ GenderAssociation + (1|Subject), family=binomial, Data)*

Random effects

| **Group** | **Name** | **Var** | ***SD*** |
| --- | --- | --- | --- |
| Subject | Intercept | 0.09 | 0.29 |
| Fragrance | Intercept | 0.11 | 0.33 |

| **Variable** | **Coeff. Est.** | ***SE*** | ***z*** | ***p*** |
| --- | --- | --- | --- | --- |
| Intercept | 0.34 | 0.23 | 1.50 | .13 |
| Gender Association | 0.65 | 0.34 | 1.94 | .05 |

Fixed effects

**S2.1.2. Grammatical gender = masculine**

*Data.lmer = glmer(Accuracy ~ GenderAssociation + (1|Subject), family=binomial, Data)*

Random effects

| **Group** | **Name** | **Var** | ***SD*** |
| --- | --- | --- | --- |
| Subject | Intercept | 0.13 | 0. 37 |

| **Variable** | **Coeff. Est.** | ***SE*** | ***z*** | ***p*** |
| --- | --- | --- | --- | --- |
| Intercept | 0.77 | 0.25 | 3.09 | < .001 |
| Gender Association | -.22 | 0.33 | -0.66 | .51 |

Fixed effects

**S2.2. How likely are you to buy this fragrance for your mother or sister?**

*Data.lmer = lmer(VasScore ~ FragranceGender*GrammaticalGender*SemanticGender + (1|Fragrance) + (1|Subject), Data)*

Random effects

| **Group** | **Name** | **Var** | ***SD*** |
| --- | --- | --- | --- |
| Subject | Intercept | 94.68 | 9.73 |
| Fragrance | Intercept | 0 | 0 |

Fixed effects

| **Variable** | **Coeff. Est.** | ***SE*** | ***t*** | ***p*** |
| --- | --- | --- | --- | --- |
| Intercept | 51.81 | 4.03 | 12.86 | < .001 |
| Fragrance Gender | -16.74 | 5.29 | -3.17 | .002 |
| Grammatical Gender | -1.19 | 5.29 | -0.23 | .82 |
| Gender Association | -19.67 | 5.29 | -3.72 | < .001 |
| Fragrance Gender*Grammatical Gender | 4.34 | 7.50 | 0.58 | .56 |
| Fragrance Gender* Gender Association | 0.98 | 7.47 | 0.13 | .90 |
| Grammatical Gender* Gender Association | -7.20 | 7.50 | -0.96 | .34 |
| Fragrance Gender*Grammatical Gender*Gender Association | 2.3 | 10.61 | 0.22 | .83 |

**S2.3. How likely are you to buy this fragrance for your father or brother?**

*Data.lmer = lmer(VasScore ~ FragranceGender*GrammaticalGender*SemanticGender + (1|Fragrance) + (1|Subject), Data)*

Random effects

| **Group** | **Name** | **Var** | ***SD*** |
| --- | --- | --- | --- |
| Subject | Intercept | 101.59 | 10.08 |
| Fragrance | Intercept | 25.44 | 5.04 |

Fixed effects

| **Variable** | **Coeff. Est.** | ***SE*** | ***t*** | ***p*** |
| --- | --- | --- | --- | --- |
| Intercept | 5.01 | 4.64 | 1.08 | .29 |
| Fragrance Gender | 15.87 | 6.18 | 2.57 | .02 |
| Grammatical Gender | 3.22 | 5.05 | 0.64 | .52 |
| Gender Association | 17.29 | 5.05 | 3.43 | < .001 |
| Fragrance Gender*Grammatical Gender | -4.73 | 7.16 | -0.66 | .51 |
| Fragrance Gender* Gender Association | -1.06 | 7.13 | -0.15 | .88 |
| Grammatical Gender* Gender Association | 13.22 | 7.16 | 1.85 | .07 |
| Fragrance Gender*Grammatical Gender*Gender Association | -11.0 | 10.14 | -1.08 | .28 |

**S2.4. How much would you pay for this fragrance?**

*Data.lmer = lmer(VasScore ~ FragranceGender*GrammaticalGender*SemanticGender + (1|Fragrance) + (1|Subject), Data)*

Random effects

| **Group** | **Name** | **Var** | ***SD*** |
| --- | --- | --- | --- |
| Subject | Intercept | 168.42 | 12.98 |
| Fragrance | Intercept | 1.29 | 1.13 |

Fixed effects

| **Variable** | **Coeff. Est.** | ***SE*** | ***t*** | ***p*** |
| --- | --- | --- | --- | --- |
| Intercept | 26.19 | 2.81 | 9.33 | <.001 |
| Fragrance Gender | -1.49 | 2.78 | -0.54 | .60 |
| Grammatical Gender | -1.0 | 2.66 | -0.37 | .71 |
| Gender Association | -3.19 | 2.66 | -1.20 | .23 |
| Fragrance Gender*Grammatical Gender | 1.48 | 3.78 | 0.39 | .70 |
| Fragrance Gender* Gender Association | 3.17 | 3.78 | 0.84 | .40 |
| Grammatical Gender* Gender Association | 4.26 | 3.78 | 1.13 | .26 |
| Fragrance Gender*Grammatical Gender*Gender Association | -4.67 | 5.36 | -0.87 | .39 |

**S2.5. How clearly could you smell the ingredients in the fragrance?**

*Data.lmer = lmer(VasScore ~ FragranceGender*GrammaticalGender*SemanticGender + (1|Fragrance) + (1|Subject), Data)*

Random effects

| **Group** | **Name** | **Var** | ***SD*** |
| --- | --- | --- | --- |
| Subject | Intercept | 181.95 | 13.49 |
| Fragrance | Intercept | 28.77 | 5.36 |

Fixed effects

| **Variable** | **Coeff. Est.** | ***SE*** | ***t*** | ***p*** |
| --- | --- | --- | --- | --- |
| Intercept | 52.74 | 4.66 | 11.33 | < .001 |
| Fragrance Gender | -11.82 | 5.89 | -2.0 | .06 |
| Grammatical Gender | -15.32 | 4.51 | -3.40 | < .001 |
| Gender Association | -20.84 | 4.51 | -4.62 | < .001 |
| Fragrance Gender*Grammatical Gender | 13.44 | 6.40 | 2.10 | 0.04 |
| Fragrance Gender* Gender Association | 12.50 | 6.37 | 1.96 | 0.05 |
| Grammatical Gender* Gender Association | 16.46 | 6.40 | 2.57 | 0.01 |
| Fragrance Gender*Grammatical Gender*Gender Association | -1.33 | 9.06 | -0.15 | 0.88 |

**Follow-up tests**

**S2.5.1. Fragrance gender = female**

*Data.lmer = lmer(VasScore ~ GenderAssociation + (1|Fragrance) + (1|Subject), Data)*

Random effects

| **Group** | **Name** | **Var** | ***SD*** |
| --- | --- | --- | --- |
| Subject | Intercept | 232.23 | 15.24 |
| Fragrance | Intercept | 26.26 | 5.13 |

| **Variable** | **Coeff. Est.** | ***SE*** | ***t*** | ***p*** |
| --- | --- | --- | --- | --- |
| Intercept | 45.08 | 4.07 | 11.07 | < .001 |
| Gender Association | -12.79 | 3.01 | -4.25 | < .001 |

Fixed effects

**S2.5.2. Fragrance gender = male**

*Data.lmer = lmer(VasScore ~ GenderAssociation + (1|Fragrance) + (1|Subject), Data)*

Random effects

| **Group** | **Name** | **Var** | ***SD*** |
| --- | --- | --- | --- |
| Subject | Intercept | 169.79 | 13.03 |
| Fragrance | Intercept | 32.59 | 5.71 |

Fixed effects

| **Variable** | **Coeff. Est.** | ***SE*** | ***t*** | ***p*** |
| --- | --- | --- | --- | --- |
| Intercept | 40.16 | 4.24 | 9.47 | < .001 |
| Gender Association | -1.24 | 3.40 | -0.36 | .72 |

**S2.5.3. Fragrance gender = female**

*Data.lmer = lmer(VasScore ~ GrammaticalGender + (1|Fragrance) + (1|Subject), Data)*

Random effects

| **Group** | **Name** | **Var** | ***SD*** |
| --- | --- | --- | --- |
| Subject | Intercept | 220.48 | 14.85 |
| Fragrance | Intercept | 25.11 | 5.01 |

| **Variable** | **Coeff. Est.** | ***SE*** | ***t*** | ***p*** |
| --- | --- | --- | --- | --- |
| Intercept | 42.32 | 4.06 | 10.42 | < .001 |
| Grammatical Gender | -7.2 | 3.17 | -2.28 | 0.02 |

Fixed effects

**S2.5.4. Fragrance gender = male**

*Data.lmer = lmer(VasScore ~ GrammaticalGender + (1|Fragrance) + (1|Subject), Data)*

Random effects

| **Group** | **Name** | **Var** | ***SD*** |
| --- | --- | --- | --- |
| Subject | Intercept | 171.5 | 13.09 |
| Fragrance | Intercept | 33.1 | 5.75 |

| **Variable** | **Coeff. Est.** | ***SE*** | ***t*** | ***p*** |
| --- | --- | --- | --- | --- |
| Intercept | 36.75 | 4.24 | 8.67 | < .001 |
| Grammatical Gender | 5.62 | 3.36 | 1.67 | 0.10 |

Fixed effects

**S2.5.5. Gender association = female**

*Data.lmer = lmer(VasScore ~ GrammaticalGender + (1|Fragrance) + (1|Subject), Data)*

Random effects

| **Group** | **Name** | **Var** | ***SD*** |
| --- | --- | --- | --- |
| Subject | Intercept | 254.6 | 15.96 |
| Fragrance | Intercept | 113.6 | 10.66 |

| **Variable** | **Coeff. Est.** | ***SE*** | ***t*** | ***p*** |
| --- | --- | --- | --- | --- |
| Intercept | 46.82 | 5.03 | 9.30 | < .001 |
| Grammatical Gender | -8.78 | 3.20 | -2.74 | 0.007 |

Fixed effects

**S2.5.6. Gender association = male**

*Data.lmer = lmer(VasScore ~ GrammaticalGender + (1|Fragrance) + (1|Subject), Data)*

Random effects

| **Group** | **Name** | **Var** | ***SD*** |
| --- | --- | --- | --- |
| Subject | Intercept | 102.2 | 10.11 |
| Fragrance | Intercept | 0.0 | 0.0 |

| **Variable** | **Coeff. Est.** | ***SE*** | ***t*** | ***p*** |
| --- | --- | --- | --- | --- |
| Intercept | 32.19 | 2.72 | 11.84 | < .001 |
| Grammatical Gender | 7.42 | 3.16 | 2.35 | 0.02 |

Fixed effects

**S2.6. How intense is this fragrance?**

*Data.lmer = lmer(VasScore ~ FragranceGender*GrammaticalGender*SemanticGender + (1|Fragrance) + (1|Subject), Data)*

Random effects

| **Group** | **Name** | **Var** | ***SD*** |
| --- | --- | --- | --- |
| Subject | Intercept | 67.00 | 8.19 |
| Fragrance | Intercept | 31.34 | 5.60 |

Fixed effects

| **Variable** | **Coeff. Est.** | ***SE*** | ***t*** | ***p*** |
| --- | --- | --- | --- | --- |
| Intercept | 65.55 | 3.82 | 17.18 | < .001 |
| Fragrance Gender | 4.55 | 5.09 | 0.89 | .39 |
| Grammatical Gender | -3.29 | 3.21 | -1.03 | .31 |
| Gender Association | -4.36 | 3.21 | -1.36 | .18 |
| Fragrance Gender*Grammatical Gender | -0.36 | 4.55 | -0.08 | .94 |
| Fragrance Gender* Gender Association | -1.76 | 4.53 | -0.39 | .70 |
| Grammatical Gender* Gender Association | 1.70 | 4.55 | 0.37 | .71 |
| Fragrance Gender*Grammatical Gender*Gender Association | 6.68 | 6.44 | 1.04 | .30 |

**S2.7. How pleasant is this fragrance?**

*Data.lmer = lmer(VasScore ~ FragranceGender*GrammaticalGender*SemanticGender + (1|Fragrance) + (1|Subject), Data)*

Random effects

| **Group** | **Name** | **Var** | ***SD*** |
| --- | --- | --- | --- |
| Subject | Intercept | 67.16 | 8.20 |
| Fragrance | Intercept | 14.11 | 3.76 |

Fixed effects

| **Variable** | **Coeff. Est.** | ***SE*** | ***t*** | ***p*** |
| --- | --- | --- | --- | --- |
| Intercept | 59.48 | 4.06 | 14.66 | < .001 |
| Fragrance Gender | -3.08 | 5.45 | -0.56 | .58 |
| Grammatical Gender | 2.01 | 4.76 | 0.42 | .67 |
| Gender Association | -2.70 | 4.76 | -0.56 | .57 |
| Fragrance Gender*Grammatical Gender | -2.30 | 6.76 | -0.34 | .73 |
| Fragrance Gender* Gender Association | -0.23 | 6.73 | -0.03 | .97 |
| Grammatical Gender* Gender Association | -4.88 | 6.76 | -0.72 | .47 |
| Fragrance Gender*Grammatical Gender*Gender Association | 0.02 | 9.57 | 0.002 | .99 |
